# Transcriptional Changes in Damask Rose Suspension Cell Culture Revealed by RNA Sequencing

**DOI:** 10.3390/plants13050602

**Published:** 2024-02-22

**Authors:** Won Kyong Cho, Hoseong Choi, Soo-Yun Kim, Euihyun Kim, Seung Hye Paek, Jiyeon Kim, Jihyeok Song, Kyoungyeon Heo, Jiae Min, Yeonhwa Jo, Jeong Hun Lee, Sang Hyun Moh

**Affiliations:** 1 College of Biotechnology and Bioengineering, Sungkyunkwan University, Suwon 16419, Republic of Korea; wonkyong@gmail.com (W.K.C.); yeonhwajo@gmail.com (Y.J.); 2 Plant Health Center, Seoul National University, Seoul 08826, Republic of Korea; bioplanths@gmail.com; 3 Plant Cell Research Institute of BIO-FD&C Co., Ltd., Incheon 21990, Republic of Korea; sykim@biofdnc.com (S.-Y.K.); ehkim@biofdnc.com (E.K.); shpaek@biofdnc.com (S.H.P.); jykim@biofdnc.com (J.K.); jhsong@biofdnc.com (J.S.); kyheo@biofdnc.com (K.H.); jamin@biofdnc.com (J.M.); jhlee@biofdnc.com (J.H.L.)

**Keywords:** rose, callus, suspension cells, RNA sequencing, transcriptome

## Abstract

Damask roses (*Rosa x damascena*) are widely used in cosmetics and pharmaceutics. Here, we established an in vitro suspension cell culture for calli derived from damask rose petals. We analyzed rose suspension cell transcriptomes obtained at two different time points by RNA sequencing to reveal transcriptional changes during rose suspension cell culture. Of the 580 coding RNAs (1.3%) highly expressed in the suspension rose cells, 68 encoded cell wall-associated proteins. However, most RNAs encoded by the chloroplasts and mitochondria are not expressed. Many highly expressed coding RNAs are involved in translation, catalyzing peptide synthesis in ribosomes. Moreover, the amide metabolic process producing naturally occurring alkaloids was the most abundant metabolic process during the propagation of rose suspension cells. During rose cell propagation, coding RNAs involved in the stress response were upregulated at an early stage, while coding RNAs associated with detoxification and transmembrane transport were upregulated at the late stage. We used transcriptome analyses to reveal important biological processes and molecular mechanisms during rose suspension cell culture. Most non-coding (nc) RNAs were not expressed in the rose suspension cells, but a few ncRNAs with unknown functions were highly expressed. The expression of ncRNAs and their target coding RNAs was highly correlated. Taken together, we revealed significant biological processes and molecular mechanisms occurring during rose suspension cell culture using transcriptome analyses.

## 1. Introduction

Rose is a woody flowering plant belonging to the genus *Rosa* and family Rosaceae [1]. Rose plants are famous for their flowers, which have variable colors, such as white, yellow, and red. The family Rosaceae consists of 90 genera and more than 3000 species, while the genus *Rosa* includes 308 species [2]. Most species in the genus *Rosa* originate from Asia, with the exception of a few species native to other continents [3].

Different species of the genus *Rosa* can be easily hybridized. Therefore, numerous rose cultivars have been developed for their flowers, and most ornamental roses are hybrids. Roses are cultivated in gardens but are also consumed as cut flowers, which are often grown in greenhouses. Moreover, the rose is famous for its oil, which contains a mixture of volatile oils that are extracted from crushed rose petals by steam distillation [4]. Rose water, known as the attar of roses, is used for several purposes, such as cosmetics, medicine, and cooking. Damask roses (*Rosa x damascena*), a rose hybrid derived from the Gallic rose (*Rosa gallica*) and musk rose (*Rosa moschata*), are commonly used to produce rose water in Iran and Europe [5].

*R. damascena* has a wide range of pharmaceutical properties, including antioxidant, anticancer, antimicrobial, antifungal, anti-inflammatory, and analgesic effects [6,7]. Therefore, extracts of *R. damascena* have been traditionally used for the treatment of chest and abdominal pain, strengthening the heart, menstrual bleeding, digestive ailments, and constipation [8]. The main chemical components of *R. damascena* are citronellol, nonadecane, geraniol, nerol, and phenyl ethyl alcohol, depending on the plant material and extraction method [8].

A callus can be defined as an unorganized cell mass that can be induced by stress, including wounding or pathogen infection [9]. Calli can be generated from a single differentiated cell that has a totipotent ability to regenerate the whole plant body [10]. Calli can be artificially generated in vitro using two plant hormones, auxin and cytokinin [11]. Therefore, calli are now being used in a wide range of research areas as well as for industrial purposes. For example, suspension plant cell cultures are initiated using calli as an efficient way to obtain plant metabolites instead of obtaining metabolites directly from mature plants, which is costly and time-consuming.

Transcriptome analysis using RNA sequencing is a popular approach for revealing the biological processes and molecular mechanisms occurring in certain tissues or conditions. Several previous studies have conducted transcriptome analyses associated with calli from various plant species, such as Arabidopsis [12], wheat [13], sorghum [13], and maize [14]. For example, embryogenic callus formation in response to salt stress in barley [15] and two *Gossypium* species [16] has been reported. Molecular differences between red and yellow–green calli of *Cyclocarya paliurus* have been revealed using RNA sequencing [17]. Moreover, two different melon transcriptomes derived from embryogenic and non-embryogenic calli were previously compared [18].

Plant genomes encode a wide range of small RNAs that play important roles in plant development, reproduction, and defense mechanisms against pathogens. The most well-known small RNAs are microRNAs (miRNAs), secondary siRNAs, and heterochromatic siRNAs, with sizes ranging from 21 to 24 nucleotides [19]. Furthermore, miRNAs and small RNAs have been identified in rice embryogenic callus [20].

In this study, we generated calli from rose petals, which are useful raw materials for the extraction of plant metabolites. Rose suspension cells were harvested at two different time points and subjected to RNA sequencing to reveal significant biological processes and molecular mechanisms during rose suspension cell culture based on transcriptional changes of coding and non-coding (nc) RNAs.

## 2. Materials and Methods

### 2.1. Rose Sample and Generation of Rose Callus

The petals of the damask rose (*Rosa x damascena*) were used to induce callus formation. First, the rose buds were washed with 70% EtOH for 30 s and sterilized with 0.3% NaClO for 20 min. The buds were then washed three times with distilled water and kept under aseptic conditions. Sterilized buds were cut and the petals were separated using a scalpel. The rose petals were then transferred to a Murashige and Skoog (MS) medium (Duchefa, Haarlem, The Netherlands) (Cat. No. M0222) containing 9.04 µM of dichlorophenoxyacetic acid (2,4-D) (Duchefa) (Cat. No. D0911) and 0.46 µM of kinetin (Duchefa) (Cat. No. K0905) and incubated in darkness at 25 °C for callus induction. The primary cultured explants were subcultured every two weeks in the same medium. The induced calli were then transferred to a new Petri dish every two weeks for callus propagation.

### 2.2. Suspension Cell Culture of Rose Callus

The selected rose callus line was used for suspension cell culture in bioreactors at the Anti-Aging Research Institute of BIO-FD&C Co., Ltd., Incheon, Republic of Korea. We conducted four different suspension subcultures of 0.5, 3, 5, and 10 L every two weeks. Finally, 2.2 kg of cells were transferred to 10 L bioreactors. Two liters of freshly prepared MS medium supplemented with 30 g/L of sucrose and 1 mg/L of 2,4-D (pH 5.8) were added to each bioreactor, and 3 independent bioreactors were used. The rose cells were further cultured for six days and eight days, respectively, in the dark. In plant cell culture, the harvesting period is typically determined after the stabilization period when the accumulation of secondary metabolites begins and before the reduction period when growth slows or stops. In this experiment, rose cells were inoculated at a density of 1.1 kg/L to reach the maximum division period 5 days after inoculation by increasing the critical density. In our study, samples were collected during the stabilization period (day 6) and the reduction period (day 8) to compare transcriptional changes. The rose suspension cells display a cell doubling time of seven days, with a growth rate of 0.0991.

### 2.3. Extraction of Total RNAs and Preparation of Libraries for RNA Sequencing

The rose cells were cultured continuously in a bioreactor, and 100 mL of the culture was sampled with the medium. After removing the medium using a nonwoven fabric, the sampled rose cells were weighed, and the cell count was determined using Laurence Lamboursain and Mario Jolicoeur’s method [21]. Cells with a mass of 1 mg were separated to the greatest extent possible and subjected to enzymatic treatment to facilitate the breakdown into smaller cellular entities, and the number of cells was measured using a hemocytometer. At two time points, six days (6D) and eight days (8D), 100 mL of cultured rose cells containing cells and liquid medium were harvested. Rose cells were isolated by centrifugation and then frozen in liquid nitrogen. Rose cells were homogenized using a mortar and pestle in liquid nitrogen. Total RNAs were extracted using the RNeasy Plant Mini Kit (Qiagen, Hilden, Germany) (Cat. No. 74904), according to the manufacturer’s instructions. The quality of the extracted total RNAs was measured using a 2100 Bioanalyzer (Agilent Technologies, Santa Clara, CA, USA). We used total RNAs with RNA integrity number (RIN) values greater than or equal to 7 for library preparation. Using the TruSeq RNA Library Prep Kit v2 (Illumina, San Diego, CA, USA) (Cat. No. RS-122-2001), according to the manufacturer’s instructions, we prepared six libraries that were further paired-end sequenced (101 bp × 2) using the Illumina HiSeq X system (Illumina). Raw RNA sequencing data were deposited in the sequence read archive (SRA) database of the National Center for Biotechnology Information (NCBI).

### 2.4. Preparation of Reference Transcriptome, Quality Trimming, and Alignment of Raw Data

To establish a reference transcriptome for rose transcriptome analyses, we used genome data of the Rosa chinensis variety ‘Old Blush’ (https://plants.ensembl.org/Rosa_chinensis/Info/Index) (accessed on 3 December 2022). The reference transcriptome is composed of four different transcriptome datasets, including 45,548 coding RNAs, 4969 non-coding (nc) RNAs, 72 mitochondrial RNAs (GenBank CM009589), and 77 chloroplast RNAs (GenBank CM009590). The raw data acquired underwent quality control, where bases with a Phred quality score below 20 and reads shorter than 50 bp were eliminated. This process was carried out using the BBDuk v.37.33 program based on the program’s user guide (https://jgi.doe.gov/data-and-tools/bbtools/bb-tools-user-guide/bbduk-guide/) (accessed on 4 December 2022). The clean raw data obtained as FASTQ files for each library were mapped onto the rose transcriptome, which includes 50,666 RNA sequences, using the BBMap program (Version 39.01) (https://jgi.doe.gov/data-and-tools/software-tools/bbtools/bb-tools-user-guide/bbmap-guide/) (accessed on 4 December 2022).

### 2.5. Calculation of Expression of Individual RNA and Identification of Differentially Expressed RNAs

The BBMap tool calculates the number of mapped reads, coverage reads per kilobase of transcript per million mapped reads (RPKM), and fragments per kilobase of transcripts per million mapped reads (FPKM) for each RNA (transcript). For the expression of individual RNA (transcript), we used the FPKM value. To identify differentially expressed RNAs, including coding RNAs and ncRNAs, the information associated with the number of reads in each RNA was subjected to DESeq2 algorithms implemented in the DEBrowser ver. 1.24.1. We compared the transcriptome obtained on eight days (8D) to that obtained on six days (6D). Using fold change > four times and adjusted *p*-value < 0.01, we selected differentially expressed RNAs.

### 2.6. Gene Enrichment Analyses

To identify enriched functions in the selected coding RNAs, we conducted gene ontology (GO) enrichment analysis using ShinyGO 0.76.3 (http://bioinformatics.sdstate.edu/go/) (accessed on 4 December 2022) against the genome of *Rosa chinensis*. The accession numbers according to the Ensembl annotation (https://asia.ensembl.org/index.html) (accessed on 4 December 2022) for the selected RNAs were subjected to GO enrichment analysis. Using an FDR < 0.5, we identified enriched GO terms in each RNA set. The top enriched GO terms were visualized using network analysis provided by ShinyGO.

### 2.7. Identification of Target Coding RNAs for ncRNAs

To identify target coding RNAs for ncRNAs, nucleotide sequences of all rose ncRNAs were subjected to a BLASTN search against all rose coding RNA sequences. Using an E-value < 1 × 10^−10^ as the cutoff, we identified potential coding RNAs that showed strong sequence similarity to the target coding RNA.

### 2.8. Generation of Interaction Network between ncRNAs and Coding RNAs

To visualize the interactions of ncRNAs with target coding RNAs, we generated an interaction network using Cytoscape ver. 3.9.1. Log2 converted fold changes for the expression of coding RNAs were also provided in the interaction network by different colors, such as upregulation (red color) and downregulation (green color).

### 2.9. Prediction of Subcellular Localization for Proteins Encoded by Rose RNAs

The protein sequences encoded by 580 coding RNAs were analyzed using TargetP ver. 2.0. (https://services.healthtech.dtu.dk/service.php?TargetP-2.0) (accessed on 5 December 2022). Based on the presence of N-terminal peptides, the proteins were categorized as secreted proteins with signal peptides (SP), mitochondrial proteins with mitochondrial transit peptides, and chloroplast proteins with chloroplast transit peptides. Proteins without N-terminal peptides were regarded as being targeted for unknown subcellular localization.

### 2.10. Quantitative Real-Time RT-PCR

Cultured calli were harvested and promptly transferred to liquid nitrogen for enhanced RNA isolation before being stored at −80 °C until RNA extraction. The RNA extraction utilized the RNeasy Plant Mini Kit (Qiagen), followed by mRNA quantification using a DeNovix Spectrophotometer (DeNovix Inc., Wilmington, NC, USA). Subsequent steps involved synthesizing complementary DNA (cDNA) with the ReverTra AceTM qPCR RT Master Mix (Toyobo, Tsuruga, Japan) as per the manufacturer’s protocol. For quantitative real-time RT-PCR (qRT-RT-PCR), the reaction mixtures comprised 10 μL of THUNDERBIRDTM Next SYBR^®^ qPCR Mix (Toyobo), 1 μL each of forward and reverse primers (10 pmol/μL and 10 pmol/mL, respectively), 7 μL of Nuclease-Free Water, and 1 μL of cDNA in a PCR plate. Amplification utilized the Rotor-Gene Q 6plex System (Rotor-Gene Q, Qiagen) involving 40 cycles: denaturation at 95 °C for 15 s, annealing at 62 °C for 1 min, and extension at 72 °C for 1 min. Each plate contained a minimum of three replications. Normalization of target gene expression was performed against the endogenous control gene (RhEF1) using the previously mentioned forward primer GGGTAAGGAGAAGGTTCACATC and reverse primer CAGCCTCCTTCTCAAACCTCT [22]. Relative expressions were calculated using the formula R = 2^−[ΔCt sample − ΔCt control]^.

## 3. Results

### 3.1. Suspension Cell Culture of Rose Callus Derived from Petals of Damask Rose and RNA Sequencing

**Figure 1:**
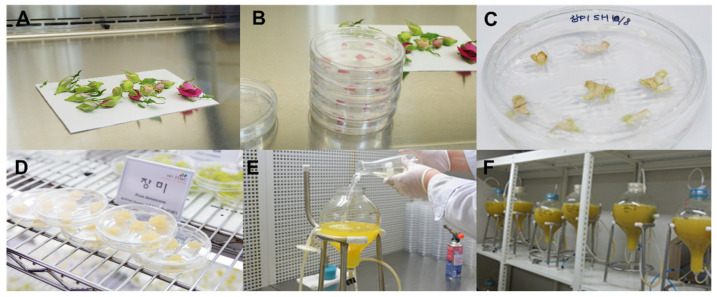
Suspension cell culture of rose callus generated from petals of damask rose. (**A**) Buds of rose were sterilized on a clean bench. (**B**) Transfer of rose petals to plates. (**C**) Induction of callus from rose petals on the medium. (**D**) Propagation of rose callus on the medium. (**E**) Transfer of subculture of rose suspension cell culture to the bioreactor. (**F**) Suspension cell culture of rose cells in dark condition.

Damask rose petals were used for callus induction (Figure 1A–D). The generated calli were transferred to a liquid medium for suspension cell culture. The subcultured suspension cells were transferred to bioreactors (Figure 1E). Rose suspension cells were propagated in the dark (Figure 1F). Three independent biological replicates were used for the suspension cell culture. The rose suspension cells were harvested at two different time points: six days (6D) and eight days (8D). Cell cultures were harvested, and total RNAs were extracted. Extracted total RNAs were used for library preparation. Finally, six libraries from the two time points were paired-end sequenced. Raw data were used for transcriptome analyses.

### 3.2. Transcriptome Analyses of Rose Suspension Cells

Raw reads were mapped to the rose reference transcriptome, which contained 50,666 RNA sequences. The amount of raw data obtained ranged from 39,300,478 reads (8D_R3) to 48,707,778 reads (6D_R3) (Table S1). The proportion of mapped reads in the reference transcriptome ranged from 94.45% (8D_R2) to 94.83% (6D_R2) (Table S1). We examined the number of mapped reads in six different transcriptome datasets (Table S2). Most of the sequenced reads were derived from coding RNAs. The proportion of reads mapped on the coding RNAs ranged from 98.04% (6D_R2) to 98.5% (8D_R2). In contrast, the number of reads mapped on ncRNAs ranged from 1.5% (8D_R2) to 1.95% (6D_R2). In addition, a very small number of reads were mapped to the mitochondrion (570 reads from six libraries) and chloroplasts (1376 reads from six libraries).

### 3.3. Expression of Coding RNAs in Rose Suspension Cells

**Figure 2:**
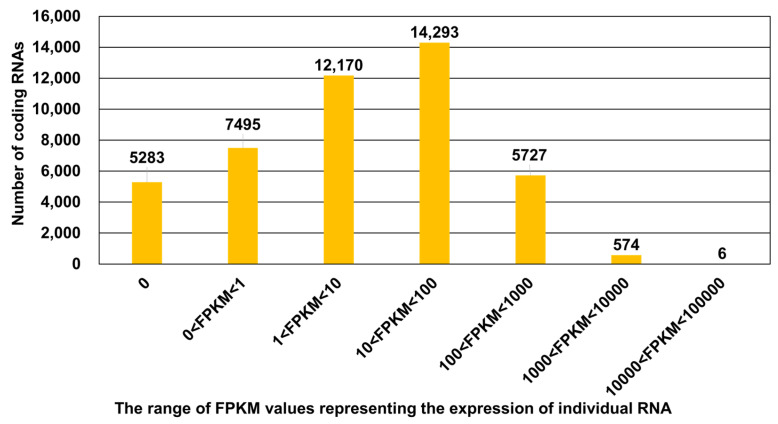
The number of coding RNAs according to expression level (FPKM value). The expression of 45,548 coding RNAs in the suspension cells was calculated based on FPKM values.

We calculated the expression levels of 45,548 coding RNAs based on FPKM values. FPKM values from six libraries were combined for each coding RNA. The 5283 coding RNAs were not expressed at all, while the FPKM values of the six coding RNAs were higher than 10,000 (Figure 2). More than half of the coding RNAs (24,948 coding RNAs) were either not expressed (FPKM = 0) or weakly expressed (FPKM < 10). The most highly expressed coding RNAs encode a glycine-rich protein, glyceraldehyde-3-phosphate dehydrogenase, protein-synthesizing GTPase, Bet v I-type allergen, translationally controlled tumor protein, and a hypothetical protein (Table S3).

**Figure 3:**
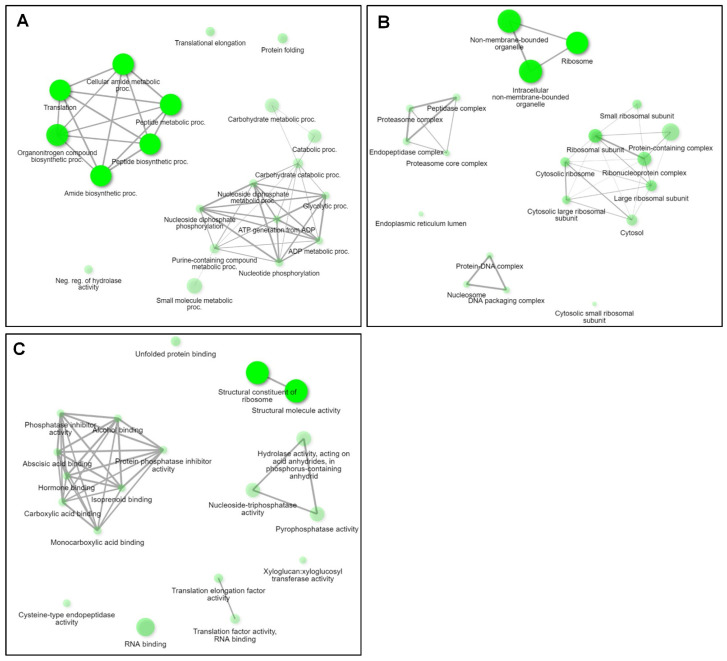
Top 20 highly enriched GO terms in 580 coding RNAs. The top 20 highly enriched GO terms in 580 coding RNAs were visualized by the network plots according to biological process (**A**), cellular component (**B**), and molecular function (**C**). The network plots display the relationship between enriched GO terms. Two GO terms (nodes) are connected if they share 20% (default) or more genes. Darker nodes are more significantly enriched gene sets. Bigger nodes represent larger gene sets. Thicker edges represent more overlapped genes. The network was generated using the ShinyGO 0.76.3 website.

We selected 580 coding RNAs with very high expression levels (FPKM > 1000) (Table S4). We examined the enriched functions of 580 coding RNAs using gene ontology (GO) enrichment analysis. We identified 165 GO terms for biological processes, 25 for cellular components, and 74 for molecular functions (Table S5). Of the enriched 165 GO terms according to biological processes, many coding RNAs were associated with translation (108 RNAs), amide biosynthesis (108 RNAs), peptide metabolism (113 RNAs), and organonitrogen compound biosynthesis (120 RNAs) (Figure 3). Moreover, GO terms for carbohydrate metabolic processes (36 RNAs), small molecule metabolic processes (45 RNAs), translation elongation (13 RNAs), and protein folding (16 RNAs) were highly enriched (Figure 3).

Out of the enriched 25 GO terms according to cellular components, three GO terms associated with non-membrane-bound organelles (125 RNAs), ribosomes (110 RNAs), and intracellular non-membrane-bounded organelles (125 RNAs) were highly enriched (Figure 3B). Ribosomal subunits (30 RNAs), protein-containing complexes (56 RNAs), ribonucleoprotein complexes (32 RNAs), large ribosomal subunits (18 RNAs), and cytosol (18 RNAs) were also identified (Figure 3B).

Of the 74 GO terms identified according to molecular function, the structural constituents of ribosomes (100 RNAs), structural molecule activity (103 RNAs), and RNA binding (55 RNAs) were significantly enriched (Figure 3C).

### 3.4. Subcellular Localization of Proteins Encoded by 580 Rose Coding RNAs

**Figure 4:**
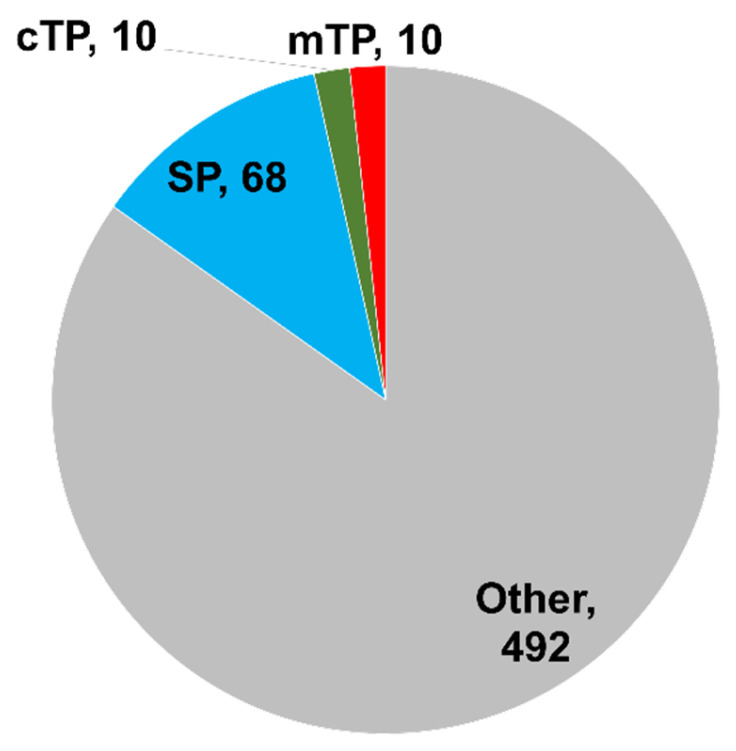
Predicted subcellular localization of 580 proteins. Subcellular localization of 580 rose proteins was predicted using TargetP ver. 2.0 based on the presence of N-terminal presequences. Secreted proteins with signal peptide (SP), a mitochondrial protein with mitochondrial transit peptide (mTP), chloroplast proteins with chloroplast transit peptide (cTP), and others indicate proteins targeted to other subcellular localizations.

Plant callus suspension cells have been used to identify secreted proteins (cell wall-associated proteins) in several proteomics studies [23,24]. The secreted proteins contain a signal peptide (SP) sequence in their N-terminal region. The protein sequences encoded by 580 rose-coding RNAs were subjected to subcellular localization prediction using the TargetP ver. 2.0. Out of 580 proteins, 68 contained signal peptides (SPs), whereas 20 possessed transit peptides (TPs) targeted to chloroplasts or mitochondria (Table S6 and Figure 4). The proteins secreted by SPs were involved in cell wall biogenesis, including the xyloglucan metabolic process (six proteins), the hemicellulose metabolic process (six proteins), the glucan metabolic process (seven proteins), the polysaccharide metabolic process (eight proteins), the carbohydrate metabolic process (10 proteins), proteolysis (10 proteins), and protein folding (four proteins).

### 3.5. Expression of ncRNAs in Rose Suspension Cells

**Figure 5:**
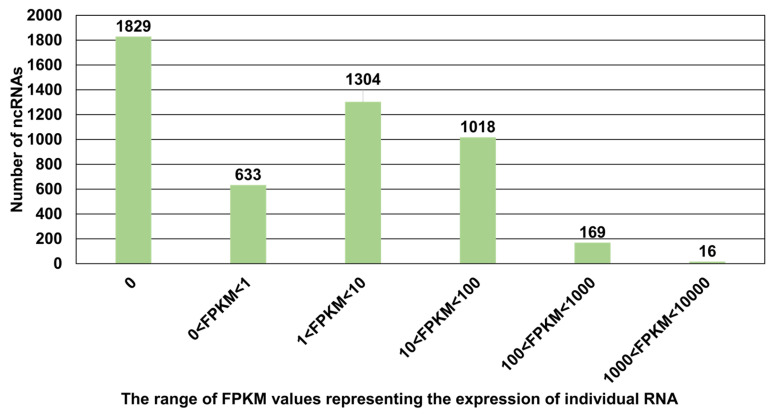
The number of ncRNAs according to expression level. The expression of 4969 ncRNAs in the suspension cells was calculated based on FPKM values.

The expression of 4969 ncRNAs was calculated. Compared with coding RNAs, the expression of ncRNAs was very low (Figure 5). For example, the FPKM value for the 1829 ncRNAs was zero (Figure 5). Moreover, about 76% of ncRNAs (FPKM < 100) were not expressed or weakly expressed. The expression of 169 ncRNAs ranged from 100 to 1000 based on FPKM values. In particular, the expression of the 16 ncRNAs was very high (Table S7). Of these, EPlT00050199787 (RchiOBHm_Chr3g0497641) encodes 18S ribosomal RNAs, while other ncRNAs do not have any known functions.

Next, we identified the target coding RNAs of the 16 ncRNAs. Of the 16 ncRNAs, 12 showed strong sequence similarity to 21 known rose coding RNAs (Figure S1). Four ncRNAs, EPlT00050201685, EPlT00050201483, EPlT00050200345, and EPlT00050199787, target a single coding RNA. EPlT00050199773 targeted three coding RNAs, PRQ44826, PRQ45980, and PRQ57720. EPlT00050200668 targets five coding RNAs. Some coding RNAs are the targets of multiple ncRNAs. For instance, PRQ18666 is the target of five ncRNAs, including lT00050199292, EPlT00050200307, EPlT00050201105, EPlT00050201527, and EPlT00050201938. We examined the functional roles of 21 coding RNAs that were targets of selected 12 ncRNAs. Of the 21 coding RNAs, 12 encoded hypothetical proteins with unknown functions. Four coding RNAs, PRQ38310, PRQ38315, PRQ38316, and PRQ38317, encode anthocyanidin 3-*O*-glucoside 5-*O*-glucosyltransferases. PRQ38482 and PRQ38489 encode G-patch domain-containing proteins. In addition, four other RNAs encode proteins associated with the 40S ribosomal protein S8, crocetin glucosyltransferase, and RNA-directed DNA polymerase. ncRNAs and their target coding RNAs were highly expressed. For instance, the FPKM value for EPlT00050200345 was 1047, whereas that for its target coding RNA (PRQ38290) was 2329.

### 3.6. Identification of Differentially Expressed RNAs between Two Different Time Points

We found that numerous RNAs were highly expressed in the rose suspension cell culture. There may be differentially expressed RNAs (DERs) during rose suspension cell culture. To this end, we compared the transcriptome of the eight days (8D) with that of the six days (6D). Based on the cutoff of a fold change (FC) > 4 and adjusted *p*-value < 0.01, we identified 680 DERs as visualized in the volcano plot (Figure S2). The 680 DERs were further divided into 390 upregulated and 290 downregulated RNAs (Figure S3A). Of the 390 upregulated RNAs, 378 were coding RNAs and 12 were ncRNAs (Table S8). Of the 290 downregulated RNAs, 258 were coding RNAs and 32 were ncRNAs (Table S9). Next, we examined the functional roles of the DERs using gene ontology (GO) term enrichment analysis. We identified 47 enriched GO terms from upregulated RNAs and 11 enriched GO terms from downregulated RNAs (Figure S3B). The number of enriched GO terms for upregulated RNAs was more than four times that of the downregulated RNAs.

In the 390 upregulated RNAs, GO terms for transmembrane transport (24 RNAs), cellular response to chemical stimulus (12 RNAs), response to toxic substance (seven RNAs), detoxification (seven RNAs), and cellular oxidant detoxification (seven RNAs) were highly enriched GO terms according to biological processes (Table S10 and Figure S4A). Moreover, we found that some upregulated RNAs were associated with phosphate ion transport (three RNAs), the photosynthesis dark reaction (two RNAs), and the response to starvation (three RNAs) (Figure S4A). According to molecular function, many GO terms in upregulated RNAs were involved in dioxygenase activity (13 RNAs), alcohol dehydrogenase (NAD+) activity (six RNAs), carbon–carbon lyase activity (eight RNAs), peroxidase activity (seven RNAs), antioxidant activity (seven RNAs), and oxidoreductase activity (seven RNAs) (Table S10 and Figure S4B).

The enriched GO terms according to biological processes in downregulated RNAs were involved in various stress responses, such as regulation of systemic acquired resistance, regulation of the immune system process, regulation of the response to biotic stimulus, regulation of the response to external stimulus, and regulation of the immune response (Table S11). Moreover, the DNA biosynthetic process (seven RNAs) and cell wall organization (seven RNAs) were also enriched in the downregulated RNAs. The downregulated RNAs encode proteins that are components of the cell wall (seven RNAs) and external encapsulating structures (seven RNAs). Many downregulated RNAs have molecular functions related to polygalacturonase activity (five RNAs).

Next, we examined coding RNAs that were targets of 12 upregulated ncRNAs. Of the 12 upregulated ncRNAs, six showed strong sequence similarity to 24 coding RNAs (Table S12 and Figure S5). Two ncRNAs, EPlT00050198817 and EPlT00050201092, may regulate seven coding RNAs (Figure S5). Of the seven coding RNAs that are targets for EPlT00050198817, three encode prenylated rab acceptor PRA1, and one is a member of the zinc finger, SWIM-type, FHY3/FAR1 family. In the case of coding RNAs for EPlT00050201092, four are members of the protein kinase RLK-Pelle-WAK family, localized on chromosome 5. The expression of most of the coding RNAs for the two ncRNAs was also highly upregulated, except for one RNA (PRQ29694) (Table S12). EPlT00050201463 regulates five coding RNAs. Of these, three (PRQ55122, PRQ58177, and PRQ19642) encode GMP synthases. However, the expression of these three RNAs was differentially regulated. For example, PRQ58177 and PRQ19642 were downregulated, whereas PRQ55122 was upregulated during suspension cell culture. EPlT00050200048 regulates three coding RNAs (PRQ33420, PRQ33440, and PRQ45464). PRQ33420 encodes 4-coumarate—CoA ligase, whereas PRQ33440 encodes AMP-dependent synthetase/ligase. Two ncRNAs (EPlT00050201641 and EPlT00050200618) regulate the expression of coding RNAs with unknown functions.

**Figure 6:**
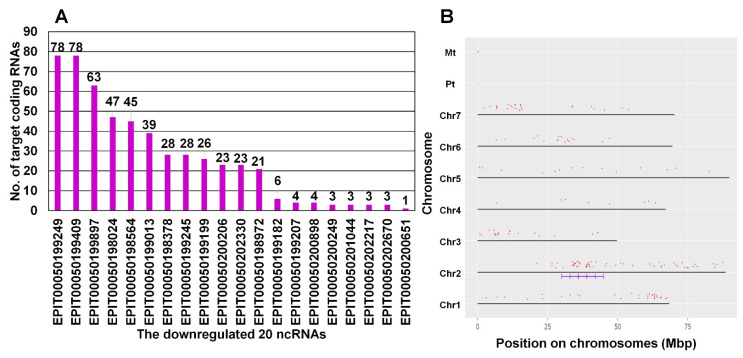
Information on target coding RNAs for the 20 ncRNAs in which expressions were downregulated. (**A**) The number of target coding RNAs for the 20 downregulated ncRNAs. (**B**) Chromosomal location of coding RNAs indicated by red dots. The enriched regions on chromosome 2 are indicated by violet lines.

The number of significantly downregulated ncRNAs was much higher than that of the upregulated ncRNAs. Moreover, the number of target coding RNAs (179 coding RNAs) for the 20 downregulated ncRNAs was much higher than that (24 coding RNAs) for the upregulated ncRNAs (Tables S12 and S13). We visualized 179 target coding RNAs for the 20 downregulated ncRNAs by network interaction using the Cytoscape program (Figure S6). We identified 526 interactions between the 20 ncRNAs and 179 coding RNAs (Table S13 and Figure S6). Interestingly, the number of target coding RNAs for the 20 downregulated ncRNAs was very high, ranging from 78 to one coding RNA (Figure 6A). The number of target coding RNAs for the 12 ncRNAs was more than 20 (Figure 6A). For instance, two ncRNAs (EPlT00050199249 and EPlT00050199409) may regulate 78 coding RNAs. Interestingly, both ncRNAs share the same list of coding RNAs. Most target coding RNAs for the two ncRNAs are hypothetical proteins with unknown functions. Some coding RNAs encode transposase-derived nuclease domain-containing protein (PRQ56094), alpha-L-fucosidase (PRQ59950), transcription factor Homeobox-WOX family (PRQ49397, PRQ49564, and PRQ49807), quinoprotein alcohol dehydrogenase-like superfamily (PRQ49445), P-loop containing nucleoside triphosphate hydrolase (PRQ50598), mediator complex subunit Med10 (PRQ24541), mitochondrial carrier protein (PRQ17477), and eukaryotic translation initiation factor 3 subunit B (PRQ20012). Based on the cutoff using fold change > 2, 14 coding RNAs that are targets of two ncRNAs were significantly downregulated. Many coding RNAs are also regulated by the identified ncRNAs. We visualized the chromosomal position of coding RNAs regulated by 20 downregulated ncRNAs (Figure 6B) and found that some coding RNAs were enriched in specific regions, such as chromosome 2.

### 3.7. Confirmation of RNA Sequencing Results by Quantitative RT-PCR

**Figure 7:**
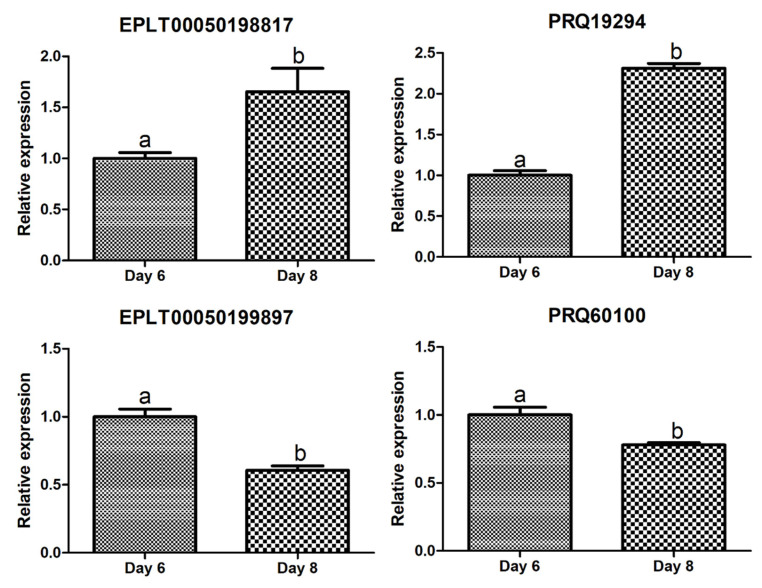
Quantitative real-time RT-PCR results of four selected RNAs. The relative expression of four selected RNAs was assessed via quantitative real-time RT-PCR and normalized using RhEF1. Three biological replicates were utilized in this analysis. Statistical significance was determined using a *t*-test. Distinct letters (e.g., “a” vs. “b”) indicate significant differences between the corresponding groups.

To validate the findings from RNA sequencing, quantitative RT-PCR (qRT-PCR) was conducted on two coding RNAs (PRQ60100 and PRQ19294) and two ncRNAs (EPLT00050199897 and EPLT00050198817) that exhibited differential expression between eight days (8D) and six days (6D) (Table S14). While PRQ19294 is annotated as a transport and Golgi organization protein, the functions of the other three RNAs remain unknown. According to the RNA sequencing data, EPLT00050198817 and PRQ19294 showed upregulation, whereas EPLT00050199897 and PRQ60100 displayed downregulation (Table S14). The qRT-PCR analysis confirmed the upregulation of EPLT00050198817 and PRQ19294, as well as the downregulation of EPLT00050199897 and PRQ60100 (Figure 7). However, the fold changes observed via RNA sequencing were notably higher than those determined by qRT-PCR. For instance, PRQ19294 exhibited approximately an eight-fold change in RNA sequencing, whereas qRT-PCR indicated a change of about 2.3 times. Importantly, all findings from both RNA sequencing and qRT-PCR yielded statistically significant results.

## 4. Discussion

The Damask rose is a famous scented rose species and an economically important rose in many industries, such as cosmetics, medicine, food, and ornamental purposes. Obtaining useful plant metabolites directly from fresh plant materials has several difficulties, such as the limitation of plant materials and prolonged cultivation time to obtain plant materials. To overcome these limitations, in vitro approaches involving tissue culture followed by suspension cell culture using bioreactors can provide a large number of plant cells for a short time at a low cost.

In this study, we established an efficient method for the large-scale production of damask rose cells. Moreover, we examined transcriptional changes in rose suspension cells by RNA sequencing. Similar to previous studies using calli, we used suspension cells, which can be frequently applied for industrial purposes. In addition, transcriptomes of suspension cells of *Bletilla striata* [25] and rosemary [26] have been recently studied by RNA sequencing. In this study, we used plant materials obtained from prolonged suspension cell cultures to reveal the molecular mechanisms underlying rose cell propagation. Moreover, we compared the transcriptomes of rose cells at two different time points to reveal the dynamic transcriptional changes during suspension cell culture.

Because of the absence of a reference transcriptome for damask roses, we used a reference transcriptome for *Rosa chinensis*. Our mapping results showed that more than 94% of the sequenced reads from *Rosa x damascena* were mapped to the transcriptome of *Rosa chinensis*. This result suggests that the two different rose species have a high degree of sequence similarity and successful application of the hetero system for transcriptome analyses. In this study, we analyzed the expression of coding RNAs and ncRNAs, as well as chloroplast and mitochondrial RNAs.

At a threshold of FPKM < 1, approximately 28% of coding RNAs were not expressed, whereas 580 coding RNAs (1.3%) were highly expressed in suspension rose cells. The data suggest that these 580 coding RNAs were the most active transcripts in suspension cells. GO enrichment analysis of 580 coding RNAs revealed that the most abundant GO terms were associated with translation and ribosomes. The ribosome is a large complex that functions as a molecular machinery to catalyze protein synthesis [27]. The high expression of RNAs encoding many proteins that are components of small and large ribosomal subunits supports the hypothesis that translation occurs intensively in rose suspension cells. Moreover, the high expression of several elongation factors in rose suspension cells indicates their functional role in facilitating translational elongation at the ribosome during protein synthesis [28]. Furthermore, a high number of coding RNAs with very high expression in rose suspension cells encode RNA-binding proteins that are involved in the synthesis, processing, transport, translation, and degradation of RNAs [29]. Therefore, strong translation activity at the ribosome in the rose suspension cells in dark conditions was highly correlated with increased biomass.

We found that highly expressed coding RNAs in rose suspension cells are involved in a wide range of metabolic pathways. Of these, peptide metabolic processes and cellular amide metabolic processes are the most abundant in rose suspension cells. Amides are used as the backbone of many peptides and play an important role in the structural units of a large number of natural and synthetic polymers [30]. They are found in a wide range of bioactive small molecules [31]. For example, amides are present in two-thirds of drug-candidate molecules [32] and are found in 25% of all pharmaceutical products available in the market [33]. Amidation reactions are regarded as the single most represented reactions in medicinal chemistry, accounting for over 50% of the processes reported in 2014 [34]. Many naturally occurring amines are also known as alkaloids, which are basic organic nitrogen compounds that are mostly derived from plants. Based on these results, we propose that many important alkaloids are synthesized in rose suspension cell culture.

Most RNAs encoded by two organelles, chloroplasts and mitochondria, were not expressed. A previous study has shown that the size and number of chloroplasts decrease in rapidly propagating callus cells [35]. Therefore, our results suggest that the functional roles of these two organelles might be decreased in suspension cell cultures. Furthermore, our libraries were generated from mRNA using oligo-dT, which specifically captures poly(A) tails. Consequently, mRNAs originating from organelles, which lack mRNAs, might exhibit lower expression levels compared with nuclear mRNAs.

We found that approximately 11% of the proteins encoding highly expressed coding RNAs were associated with cell walls, based on the presence of a signal peptide. Numerous studies have shown that cell wall-associated proteins are enriched in the plant calli. During suspension cell culture in the dark, rose callus cells were dramatically propagated without any further differentiation into other tissues. Coding RNAs associated with cell wall proteins should be highly expressed to generate plant cells at the beginning of cell culture in a bioreactor. However, as the cell mass increases in a bioreactor, the required elements in the bioreactor should be shortened. Therefore, we assumed that several cell wall-associated coding RNAs were downregulated in the late stage of rose suspension cell culture.

Of the several known stressors, wounding is the primary factor that triggers callus formation by activating cell proliferation and promoting cellular reprogramming [36]. At the initial callus formation, it has been known that stress-responsive or hormone-associated genes were highly induced [14,36]. Similarly, we also found that several stress response coding RNAs including transcription factors were induced at the early stage of rose suspension cell culture. However, their expression was gradually downregulated at the late stage of rose suspension cell culture. In contrast, the coding RNAs associated with detoxification and transmembrane transport were upregulated. Reactive oxygen species (ROS) such as superoxide and hydrogen peroxide are toxic cellular metabolites that can be detoxified by various enzymes [37]. ROS also play an important role in regulating important biological processes by acting as signaling molecules [38]. The seven detoxification-associated RNAs encode NAD(P)H oxidase (two RNAs), peroxidase (four RNAs), and a phospholipid-hydroperoxide glutathione peroxidase (one RNA). Plant NAD(P)H oxidases catalyze the production of superoxide, a type of ROS [39]. Peroxidases in plants are heme-containing monomeric glycoproteins that use hydrogen peroxide or oxygen to oxidize a wide range of molecules [40]. Phospholipid hydroperoxide glutathione peroxidases regulate ROS levels by detoxifying ROS [41]. Our results suggest that the upregulation of detoxification-associated RNAs is highly correlated with strong detoxification activities of these enzymes by regulating ROS levels produced from rose suspension cell culture.

Plant transcriptomes possess many ncRNAs, such as small RNAs and long ncRNAs (lncRNAs), which do not have protein-coding capacity but are functional. With the rapid development of high-throughput sequencing, several ncRNAs have been identified in various plant species. Plant ncRNAs may participate in numerous biological processes, such as development and stress responses by regulating gene expression [42]. We found that most ncRNAs were not expressed in rose suspension cells, but few ncRNAs were highly expressed. However, the functional roles of most ncRNAs have not yet been characterized. Therefore, we examined the target coding RNAs of the identified ncRNAs. Half of the target coding RNAs for the 12 ncRNAs that were highly expressed in rose suspension cells encoded hypothetical proteins. However, four coding RNAs regulated by ncRNAs encode anthocyanidin 3-O-glucoside 5-O-glucosyltransferases, which are involved in anthocyanin biosynthesis [43]. This result suggests that ncRNAs may regulate the expression of genes involved in anthocyanin biosynthesis in rose suspension cells.

We identified a few ncRNAs that were differentially expressed between the two time points. However, the number of target coding RNAs (179 coding RNAs) for downregulated ncRNAs was very high compared with that for upregulated ncRNAs (24 coding RNAs). Interestingly, the identified downregulated ncRNAs regulated the same set of coding RNAs. Thus, these ncRNAs may be transcribed from the same gene family composed of a large number of genes. We found that specific chromosomal regions were enriched with ncRNA-regulated coding RNAs. In addition, we showed that the expression of ncRNAs and their target RNAs were highly correlated. Unfortunately, most coding RNAs regulated by ncRNAs encode hypothetical proteins with unknown functions. Therefore, it might be of interest to examine the possible roles of these ncRNAs and their target coding RNAs in the near future.

Our results showed that many coding RNAs were differentially expressed between days six and eight.

## 5. Conclusions

In this study, we generated calli derived from the petals of damask rose, which are useful raw materials for cosmetics and medicine. Subculture of rose callus in suspension cell culture supplemented with sugar in the dark significantly promoted the growth of plant cell mass. We analyzed transcriptomes derived from rose suspension cells harvested at two different time points (6 and 8 days) by RNA sequencing. More than 94% of raw reads were mapped to the reference rose transcriptome. We identified 580 coding RNAs that were highly expressed in the rose suspension cells. These coding RNAs were associated with translation and ribosomes, indicating that strong translation activity at ribosomes in suspended rose cells is highly correlated with increased biomass. Moreover, coding RNAs involved in peptide metabolic and amide biosynthetic processes were highly expressed. Our results suggest that many proteins and amines, known as alkaloids, are synthesized in rose suspension cell culture. We found that approximately 11% of the proteins encoding highly expressed coding RNAs were associated with the cell wall. Several stress response coding RNAs including transcription factors were induced at the early stage of rose suspension cell culture, but their expression was gradually downregulated at the late stage of rose suspension cell culture. In contrast, coding RNAs associated with detoxification and transmembrane transport were upregulated in the late stage of rose suspension cell culture. We found that most ncRNAs were not expressed in rose suspension cells, but few ncRNAs were highly expressed. However, the functional roles of most ncRNAs have not yet been characterized. In addition, we showed that the expression of ncRNAs and their target RNAs were highly correlated. Most coding RNAs regulated by ncRNAs encode hypothetical proteins with unknown functions. Taken together, we revealed that RNAs involved in translation and metabolic processes were highly expressed during rose suspension cell culture, resulting in the propagation of the rose cell mass.

## Data Availability

Raw data were deposited in the NCBI SRA database with the following accession number: PRJNA916753.

## Supplementary Materials

The following are available online at https://www.mdpi.com/article/10.3390/plants13050602/s1, Figure S1: Interaction network of the 12 ncRNAs with 21 coding RNAs. The 21 coding RNAs, which are targets of the selected 12 ncRNAs, were visualized by interaction network using Cytoscape ver. 3.9.1. The ncRNAs are indicated by rectangles with orange color whereas coding RNAs are indicated by ovals with pale green color. Figure S2: Volcano plot displaying differentially expressed RNAs. The plot shows the RNA fold change on the x-axis against the adjusted *p*-value plotted on the y-axis. Figure S3: Number of DEGs and enriched GO terms. (A) The number of ncRNAs and coding RNAs in identified DEGs according to upregulated and downregulated RNAs. (B) The number of enriched GO terms in the upregulated and downregulated RNAs. Figure S4: Enriched GO terms according to biological process (A) and molecular function (B) in upregulated coding RNAs. (A) The number of ncRNAs and coding RNAs in identified DEGs according to upregulated and downregulated genes. (B) The number of enriched GO terms in the upregulated and downregulated genes. Figure S5: Interaction network of the six upregulated ncRNAs with their target coding RNAs. The 24 coding RNAs, which are targets of the selected six ncRNAs, were visualized by interaction network using Cytoscape ver. 3.9.1. The ncRNAs are indicated by a rectangle with a dark blue color whereas the coding RNAs are indicated by an oval. The log2 converted fold changes of coding RNAs were indicated by green colors for downregulated RNAs and red colors for upregulated RNAs. Figure S6: Interaction network of 20 downregulated ncRNAs with their target coding RNAs. The 179 coding RNAs, which are targets of the selected 20 ncRNAs, were visualized by an interaction network using Cytoscape ver. 3.9.1. ncRNAs are indicated by rectangles with dark blue color, whereas coding RNAs are indicated by ovals. The log2 converted fold changes of coding RNAs are indicated by green for downregulated RNAs and red for upregulated RNAs. Table S1: Summary of mapping results on the rose reference transcriptome. Table S2: The number of mapped reads on the four different rose transcriptome datasets. Table S3: Information on six coding RNAs showing high expression in rose suspension cells. Table S4: Information on the 580 coding RNAs that were highly expressed in the rose suspension cells. Table S5: Enriched GO terms in the 580 coding RNAs that were highly expressed in the rose suspension cells. Table S6: Predicted subcellular localization of proteins encoded by the 580 coding RNAs. Table S7: Information on the 16 ncRNAs that were highly expressed in the rose suspension cells. Table S8: Information on the 390 upregulated RNAs. Table S9: Information on the 290 downregulated RNAs. Table S10: Enriched GO terms for the 390 upregulated RNAs. Table S11: Enriched GO terms in downregulated coding RNAs. Table S12: Information on the 24 upregulated ncRNAs and their target coding RNAs. Table S13: Information on the 20 downregulated ncRNAs and their target coding RNAs. Table S14: Information on four selected RNAs for quantitative RT-PCR.
