# Supplementary figures and images for: Transcriptional Changes in Damask Rose Suspension Cell Culture Revealed by RNA Sequencing
Source: Plants (Basel). 2024 Feb 22;13(5):602. doi: 10.3390/plants13050602 (PMC10934739; doi:10.3390/plants13050602)

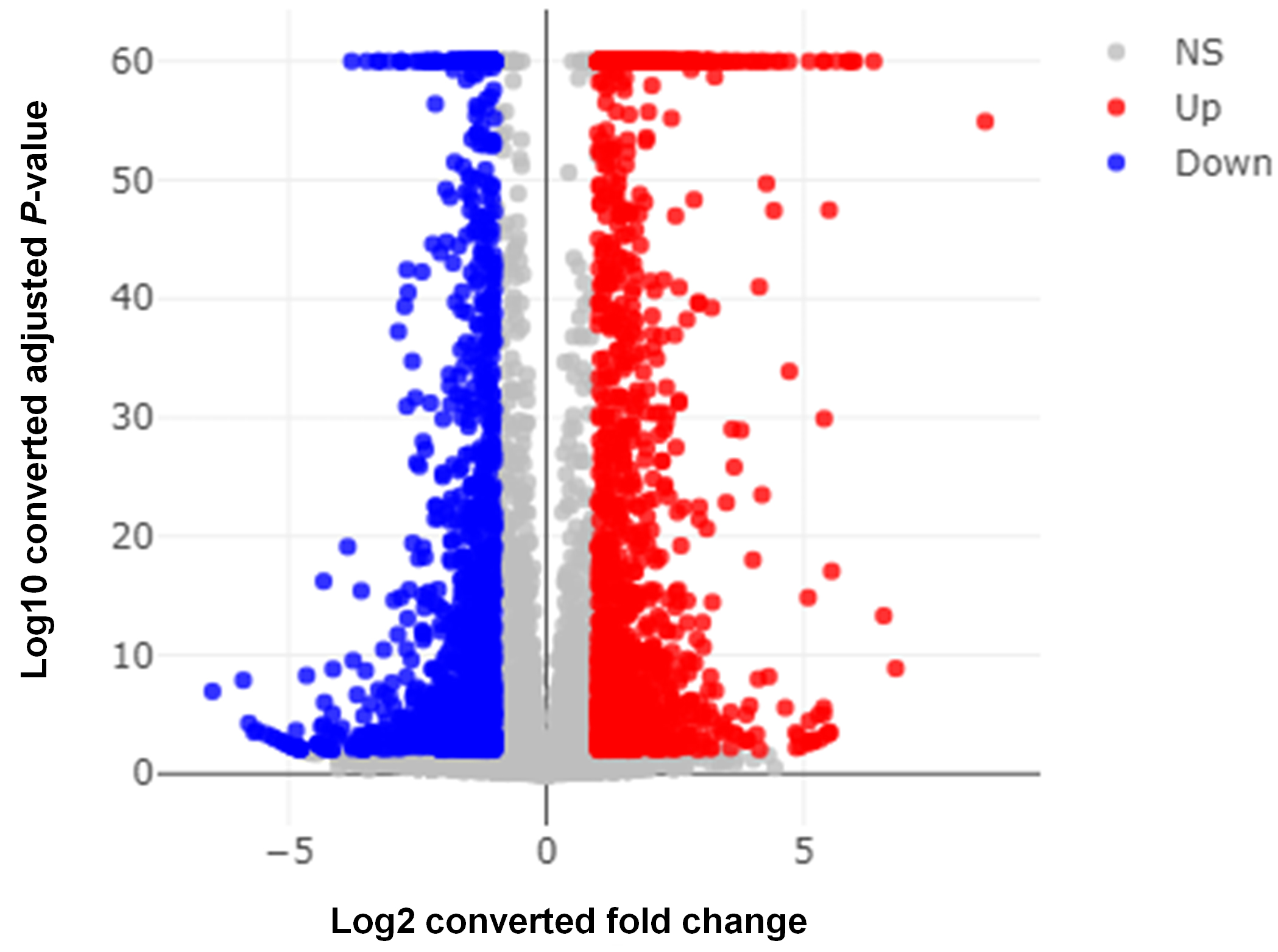

Supplement: Supplementary file 1 [file plants-13-00602-s001.zip › Figure S2.jpg]

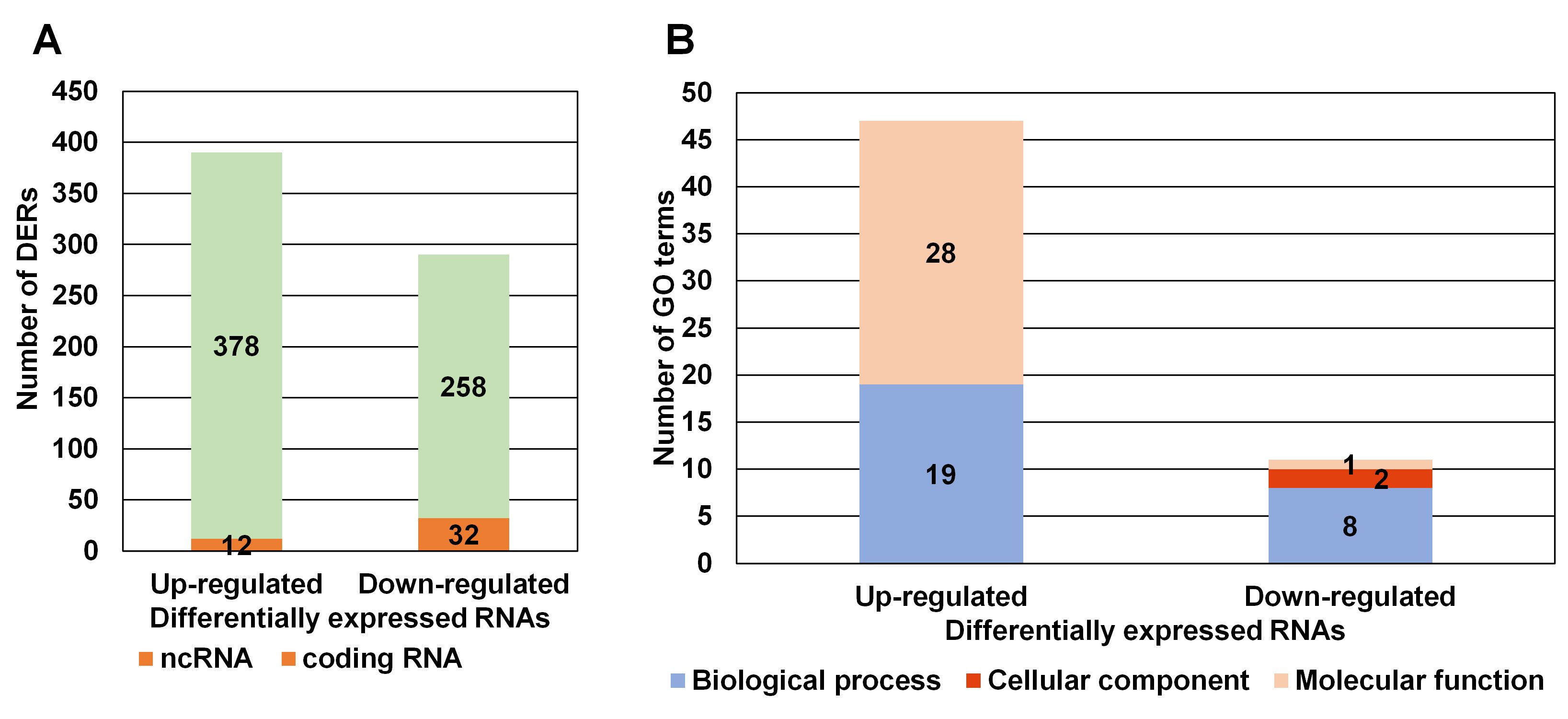

Supplement: Supplementary file 1 [file plants-13-00602-s001.zip › Figure S3.jpg]

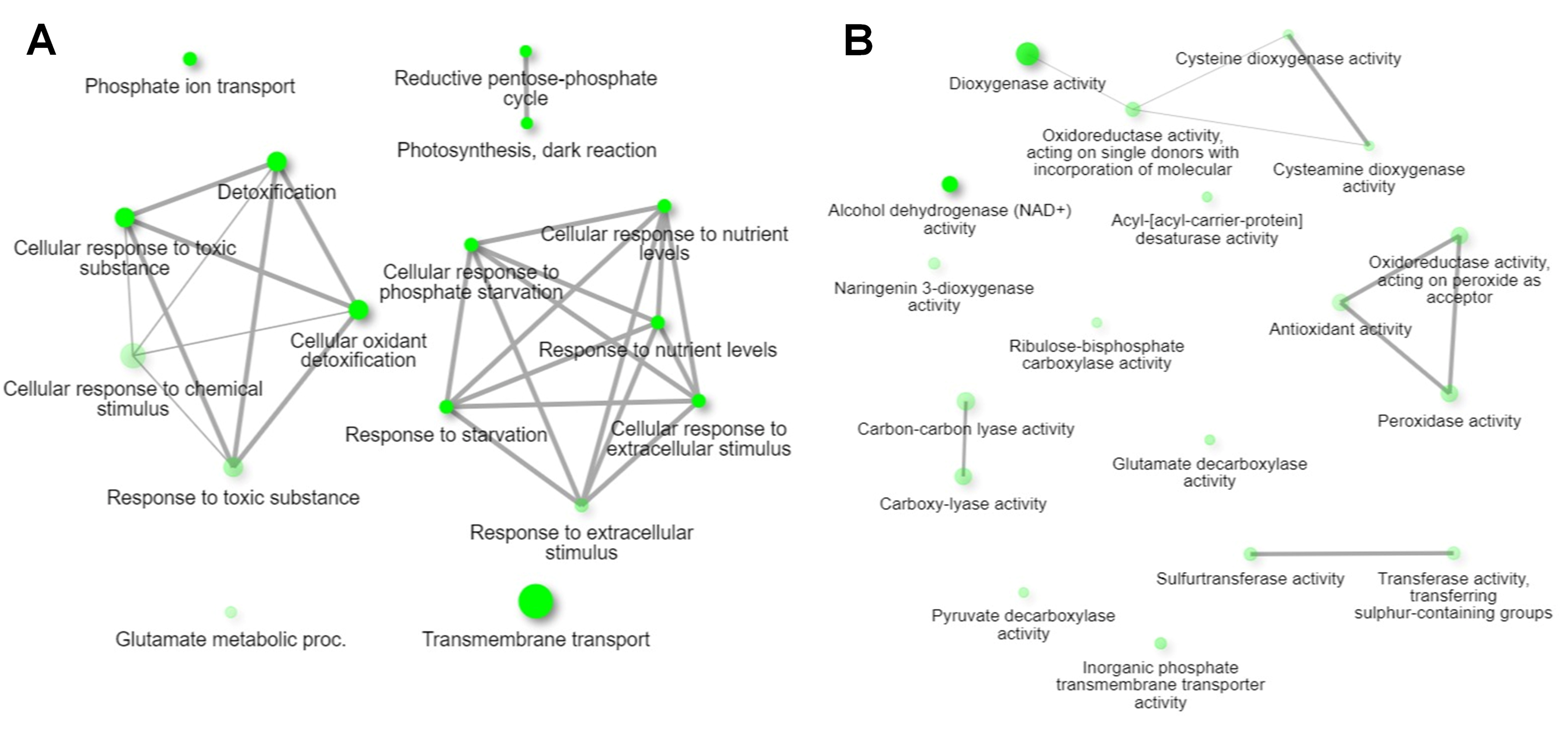

Supplement: Supplementary file 1 [file plants-13-00602-s001.zip › Figure S4.jpg]

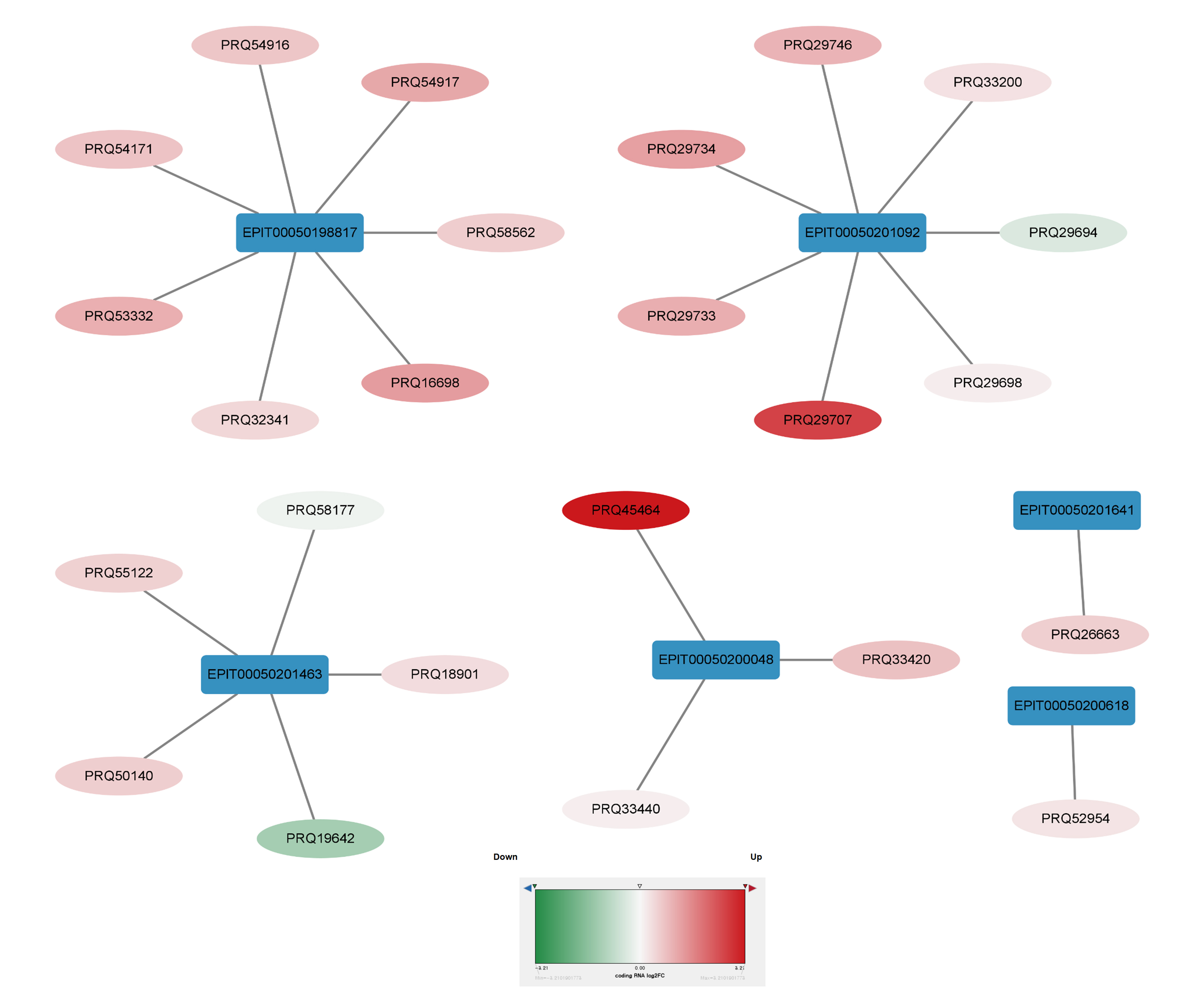

Supplement: Supplementary file 1 [file plants-13-00602-s001.zip › Figure S5.jpg]

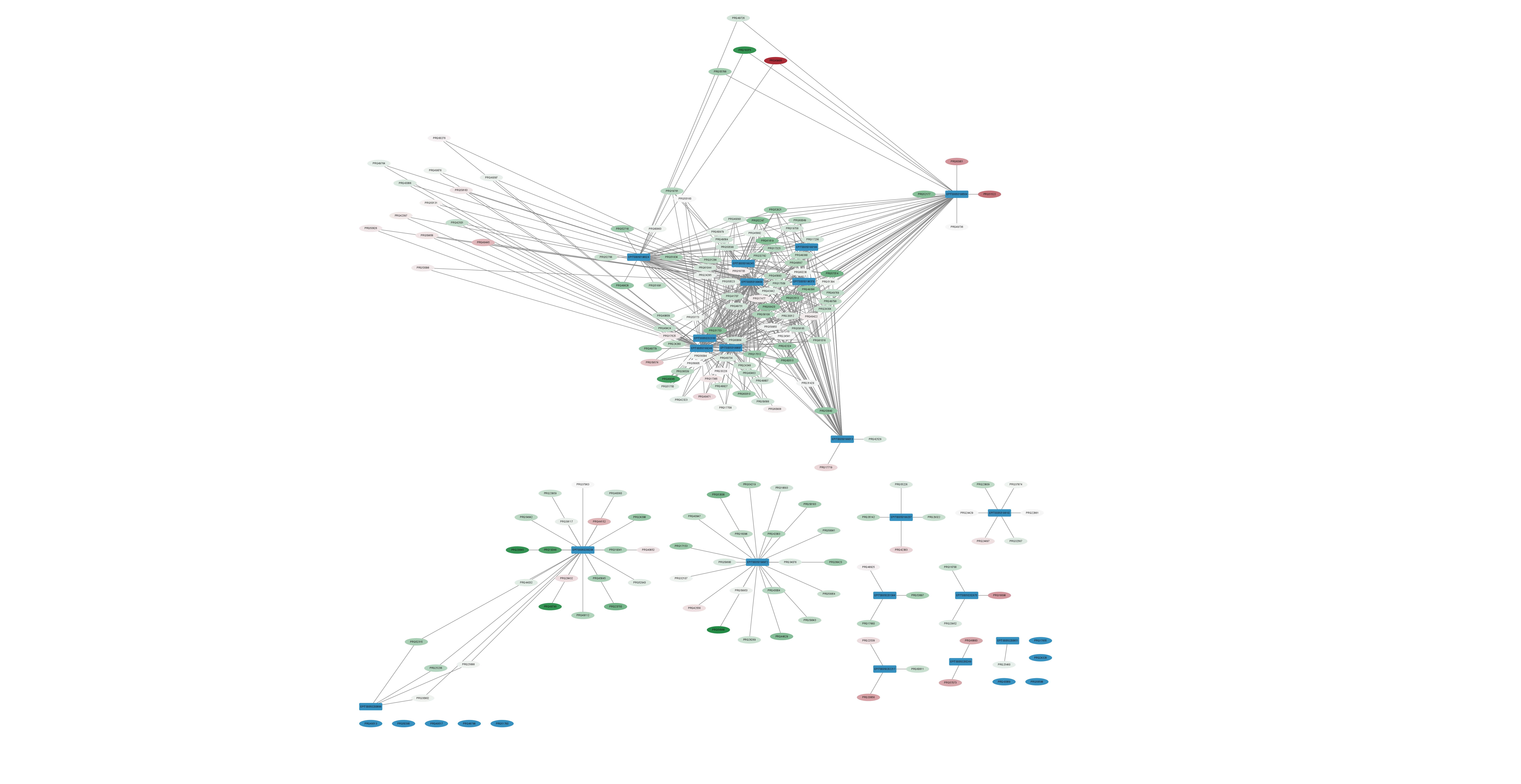

Supplement: Supplementary file 1 [file plants-13-00602-s001.zip › Figure S6.png]

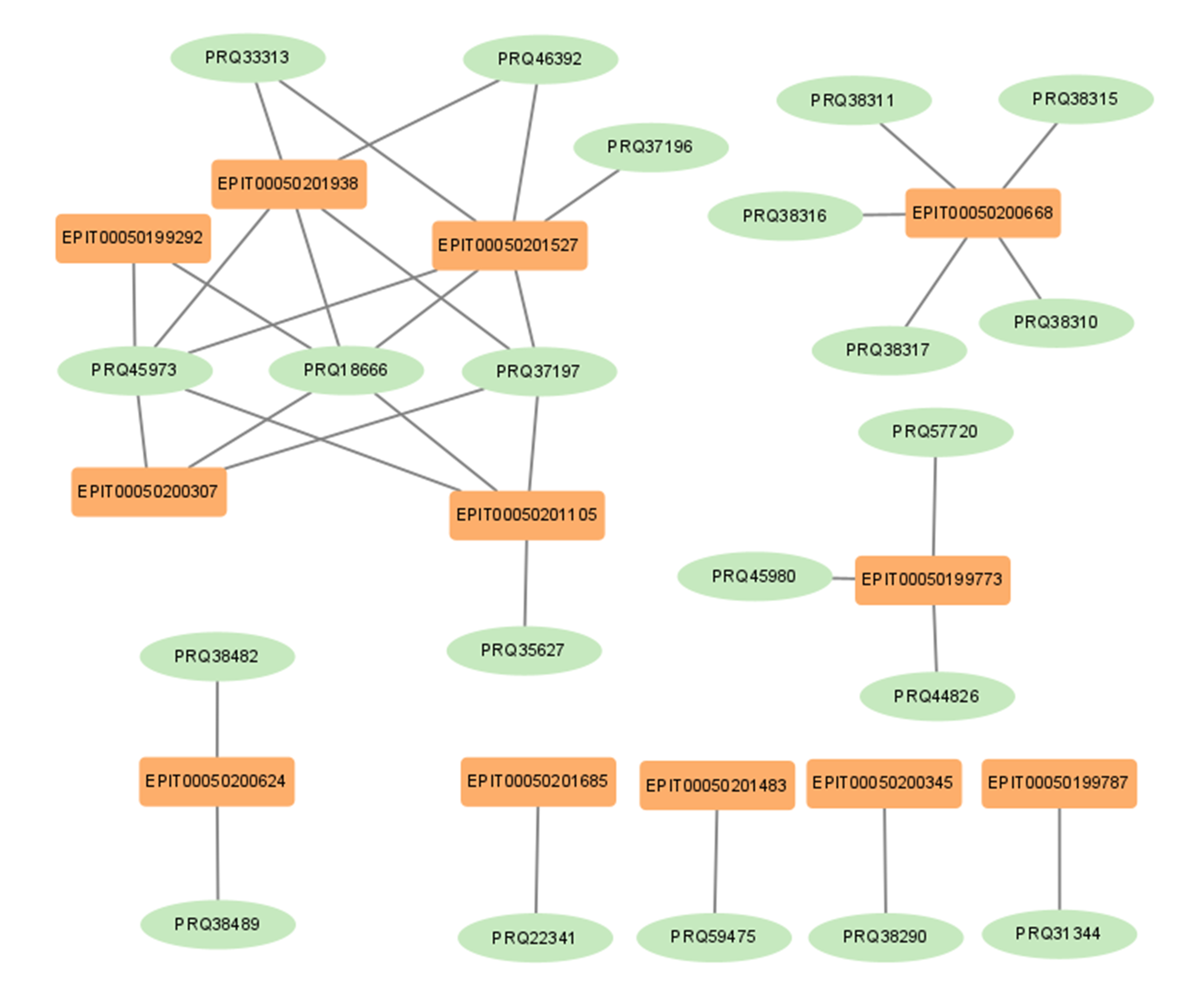

Supplement: Supplementary file 1 [file plants-13-00602-s001.zip › Figure S1.jpg]
